# High prevalence of a host‐associated *A* *naplasma* lineage in wild coatis ( *N* *asua nasua*) from the Brazilian Atlantic Forest

**DOI:** 10.1111/mve.70074

**Published:** 2026-04-18

**Authors:** Matheus Dias Cordeiro, Paulo César Magalhães‐Matos, Isadora dos Santos Dias, Jônathan David Ribas Chagas, Olívia Zen Gianfrancisco, Ellen Meireles Brandão, Claudia Bezerra da Silva, Bruna de Azevedo Baêta

**Affiliations:** ^1^ Veterinary Medicine Post Graduation Program Federal Rural University of Rio de Janeiro – UFRRJ Seropédica Brazil; ^2^ Federal Institute of Amapá‐Campus Agrícola de Porto Grande Porto Grande Brazil; ^3^ Veterinary Sciences Post Graduation Program Federal Rural University of Rio de Janeiro – UFRRJ Seropédica Brazil

**Keywords:** *Anaplasma*, *Nasua nasua*, tick‐borne pathogens, vector–host interaction, wildlife epidemiology

## Abstract

This study aimed to detect the DNA of *Anaplasma* spp., *Ehrlichia* spp., *Borrelia* spp. and protozoa of the order Piroplasmida in blood samples from 73 coatis captured at three sites in the Iguaçu National Park. PCR assays revealed a high prevalence of *Anaplasma* spp., with 47.9% (35/73) of animals testing positive for the 16S rRNA and 23S rRNA genes. None of the samples tested positive for *Borrelia* spp., *Ehrlichia* spp. or protozoa of the order Piroplasmida. Sequencing analyses indicated that all positive samples were genetically identical and closely related to *Anaplasma platys* and lineages previously described in cattle and ticks. Phylogenetic analyses based on multiple loci revealed distinct clades, supporting the circulation of a unique *Anaplasma* variant in coatis. Comparisons with tick‐derived sequences showed genetic divergence, suggesting distinct strains infecting hosts and vectors. These findings provide novel insights into the epidemiology of haemoparasites in wildlife and highlight the role of coatis as potential reservoirs of diverse *Anaplasma* lineages in the Atlantic Forest.

## Graphical abstract

- *Anaplasma* sp. DNA was detected in 47.9% of free‐ranging coatis (*Nasua nasua*) sampled in Iguaçu National Park, southern Brazil.
- Molecular analyses revealed a genetically distinct *Anaplasma* lineage infecting coatis, differing from *Anaplasma* strains detected in associated tick populations.
- No association was observed between Anaplasma infection in coatis and tick species infestation, suggesting independent ecological cycles for hosts and ticks.

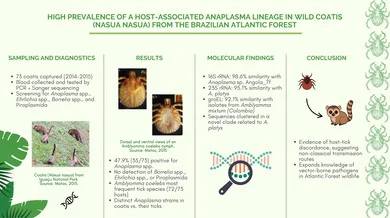

## INTRODUCTION

The Iguaçu National Park (PNI) is one of the largest continuous areas of the Atlantic Forest in Brazil and harbours populations of coatis (*Nasua nasua*), omnivorous mammals of the family Procyonidae, widely distributed throughout South America. These socially organized animals, frequently observed in tourist areas, have become a symbol of the local fauna (Magalhães‐Matos et al., 2022; Moraes et al., 2019).

The opportunistic behaviour of coatis favours their presence in places with intense human activity, such as residential areas and sites frequented by domestic animals, especially dogs and cats (Beisiegel & Mantovani, 2006; Magalhães‐Matos et al., 2022). This proximity increases the risk of interaction with pathogen vectors, requiring management strategies that reconcile biodiversity conservation with visitor safety (Araújo et al., 2023).

Several tick species have been recorded parasitizing coatis in Brazil, including *Amblyomma brasiliense*, *Amblyomma ovale*, *Amblyomma rotundatum*, *Amblyomma sculptum*, *Amblyomma parvum*, *Amblyomma coelebs*, *Amblyomma dubitatum*, *Rhipicephalus microplus*, *Rhipicephalus sanguineus* and *Haemaphysalis juxtakochi*, with occurrences documented in different Brazilian states (Barros & Baggio, 1992; Figueiredo et al., 1999; Labruna et al., 2005; Rodrigues et al., 2006; Dantas‐Torres et al., 2010; Sousa et al., 2017; Magalhães‐Matos et al., 2022; Perles et al., 2023; Perles, de Macedo, et al., 2022a).

The relevance of these ectoparasites stems from their role as vectors of pathogens capable of infecting the blood cells of multiple hosts, with potential impacts on animal and human health, as well as ecological and economic repercussions (Perles et al., 2023).

In the PNI, previous analyses have identified the circulation of *Anaplasma*, *Theileria*, *Borrelia*, *Hepatozoon*, *Mycoplasma* and *Bartonella* in coatis and associated ticks (Araújo et al., 2023; Perles et al., 2023). In this context, the present study aimed to screen for infections by bacterial species of *Anaplasma* spp., *Ehrlichia* spp., *Borrelia* spp. and protozoa of the order Piroplasmida, and to evaluate their co‐occurrence with tick infestation in coatis from the PNI.

The present study is a continuation of analyses of coatis samples carried out in the PNI, located in the municipality of Foz do Iguaçu, state of Paraná, southern Brazil. Blood samples were collected in September 2014 and March and April 2015 from coatis in three sampling sites located in the tourist area as previously described (Araújo et al., 2023; Magalhães‐Matos et al., 2017; Magalhães‐Matos et al., 2022).

Blood samples from coatis were collected from the jugular vein, stored in tubes (5 mL) containing Ethylenediaminetetraacetic acid (EDTA) anticoagulants, kept in isothermal boxes with ice and transported to the laboratory for processing.

The total DNA was extracted from the blood samples using a DNeasy Blood & Tissue Kit (QIAGEN, Hilden, Germany), following the manufacturer's instructions. The quality of blood‐derived DNA was verified by PCR amplification of the mammalian GAPDH gene using the primer set GAPDH‐F/GAPDH‐R (Table S1, File S1). Tick DNA was extracted individually using the phenol–chloroform method, quantified and assessed for purity using spectrophotometry (NanoDrop™ 2000/2000c) and standardized to 50 ng/μL for molecular analyses (Magalhães‐Matos et al., 2022). Tick DNA quality and PCR suitability were confirmed by amplification of a partial fragment of the mitochondrial 16S rRNA gene, following the original protocol (Table S1). These endogenous controls were used to assess DNA integrity and to detect potential PCR inhibitors, thereby minimizing the risk of false‐negative results.

PCR assays were performed for the initial screening of bacterial and protozoan DNA, including members of the family Anaplasmataceae (16S rRNA and 23S rRNA genes), *Borrelia* spp. (flagellin B gene), and protozoa of the order Piroplasmida (18S rRNA gene). For confirmation and molecular characterization, additional targets were used, including 16S rRNA, *gro*EL, *glt*A, *rpo*B (*Anaplasma* spp.) and *dsb* (*Ehrlichia* spp.) genes. All primers and PCR conditions followed previously published protocols and are listed in Table S1. Detailed molecular protocols applied to tick DNA are available in Araújo et al. (2023), which analysed the same tick specimens examined in the present study. For each reaction, DNA samples from *Borrelia anserina* strain AL (culture), *Babesia bigemina* (bovine positive), *Anaplasma platys* (dog positive) or *Ehrlichia canis* (dog positive) were used as positive controls, and ultrapure water was used as a negative control.

PCR products were subjected to electrophoresis in a 1.5% agarose gel, separated using electrophoresis, stained with ethidium bromide and visualized using an ultraviolet light transilluminator. PCR products were purified with Exo‐Sap‐IT (GE Healthcare®), following the manufacturer's protocol. The fragments were sequenced in both directions with an automated genetic analyser (ABI 3730 DNA Analyzer, Thermo Fisher Scientific) using Sanger sequencing with BigDye Terminator v3.1 chemistry. The obtained sequences were aligned using the DNA Baser® v5.11.3 programme and subjected to homology searches against sequences deposited in GenBank using the BLASTn tool.

Homologous sequences of 16S rRNA (*Anaplasma marginale* KU686793 and AF309866; *Anaplasma capra* MG869510 and LC432114; *Anaplasma ovis* AF414870 and JN558818; *Anaplasma* sp. MT019536, MT019537, MT019545, MT019560 and PQ571828; *An. platys* KT982643 and KX792089; *Anaplasma bovis* HM131218 and GU937020; *Anaplasma phagocytophilum* AB196721, AB196720), 23S rRNA (*An. platys* CP046391; *An*. *phagocytophilum* NC_021879 and NZ_CP166491; *An. ovis* CP015994; *An*. *marginale* NR_076579; *Anaplasma centrale* NR_076686, *Anaplasma* sp. PX804143 and PX804142), *glt*A (*An*. *platys* PV539462 and AB058782; *Candidatus* Anaplasma turritanum MW296141 and MW296140; *Candidatus* Anaplasma camelii PV797604 and PV797602; *Anaplasma* sp. JN055362, OP080693 and OP777464; *An*. *phagocytophilum* KP861637 and JQ622146; *An*. *marginale* KU586326 and AF304140; *An*. *centrale* AF304141; *An*. *capra* MW428305 and MW428304) and groEL genes (*An*. *platys* OR935806, MW450814 and EU516386; Ca. An. camelii PV797578; *An*. *capra* MZ222248; *An*. *ovis* FJ460441; *An*. *marginale* KY523038 and KY523002; *An*. *centrale* KY522998; *An*. *phagocytophilum* MZ348301 and MZ348295; *Candidatus* Anaplasma boleense KX987391), previously published in other studies were retrieved in FASTA format and aligned with the sequences obtained in this study using the ClustalW multiple alignment algorithm implemented in MEGA v10.2 software (Kumar et al., 2018). The alignment was subsequently trimmed.

The outgroup sequences used were *Neorickettsia risticii* strain Illinois (NR076635) for the 23S rRNA gene, *Rickettsia belli* (MT174170) for the *gltA* gene and *Ehrlichia ruminantium* strain Vosloo (DQ647011) for the *groEL* gene. Phylogenetic trees were inferred using the maximum likelihood (ML) method in MEGA v10.2 (Kumar et al., 2018), with the best‐fitting evolutionary model selected according to the Akaike information criterion (AIC). Node support was assessed by bootstrap analysis with 1000 replicates under a heuristic search. Final tree editing and visualization were performed using Inkscape v1.3.2. Data on tick infestation of coatis were obtained from Araújo et al. (2023), including tick species identification per animal. The association between PCR positivity and variables such as host sex, age class and tick species was evaluated using descriptive analysis. This analysis was based on the distribution of tick taxa among positive and negative hosts and is presented in Table S2.

A total of 73 coatis were evaluated in this study, of which 35 (47.9%) tested positive for *Anaplasma* spp. 16S rRNA gene by PCR. No samples showed amplification for *Borrelia* spp., *Ehrlichia* spp. or protozoans of the order Piroplasmida in the initial screening PCR assays. All samples amplified the constitutive GAPDH gene, confirming DNA integrity and thereby excluding false‐negative results due to DNA extraction failures.

Among the positive samples for the initial 16S rRNA screening, all were also confirmed positive for the *Anaplasma* spp. 23S rRNA gene. Of these, only 10 samples amplified the *gltA* and *groEL* genes, and none amplified the *rpoB* gene.

With respect to host demographics, 23 out of 49 females (46.9%) and 12 out of 26 males (46.2%) were positive for *Anaplasma* spp. Tick infestation data showed that *Am. coelebs* was the predominant tick species, infesting 72 of the 73 animals. Other tick species included *Am. ovale* (14 animals), *Amblyomma* spp. (11 animals), *Am. brasiliense* (36 animals) and *Haemaphysalis* spp. (4 animals).

Blood‐derived DNA from animals infested by *Am. coelebs* ticks previously identified as *Anaplasma* positive (Araújo et al., 2023) did not amplify the *Anaplasma* spp. 16S rRNA gene, indicating that the strains detected in ticks differ from those infecting coatis.

No clear association was observed between *Anaplasma* sp. positivity in coatis and the tick species collected, as the distribution of tick taxa was similar among positive and negative hosts.

Sequencing analyses revealed that all *Anaplasma* spp. positive samples were genetically identical across all genes analysed. The 16S rRNA sequences showed 98.62% similarity (860/872 bp) with *Anaplasma* sp. lineage Angola_7f detected in cattle in Angola (MN401148). Similarity with *An. platys* S3 strain (CP046391) was 95.13% (645/678 bp) for the 23S rRNA gene, and with *Anaplasma* sp. isolates from *Amblyomma mixtum* in Colombia was 92.12% (505/548 bp) for the *groEL* gene. The *gltA* gene sequences showed 97.84% similarity (90/92 bp) with *Anaplasma* sp. detected previously in coatis at PNI (Perles et al., 2023).

Comparative analyses between sequences obtained from coati blood and from ticks revealed sequence similarities of 97.49% (815/836 bp) for 16S rRNA and 90.22% (461/511 bp) for 23S rRNA genes, supporting the presence of genetically distinct *Anaplasma* lineages infecting coatis and their ticks.

The datasets generated and analysed during the current study are available in the NCBI GenBank database (https://www.ncbi.nlm.nih.gov/genbank/) and can be accessed through the following accession numbers: PX915630–PX915661 for 16S rRNA, PX913173–PX913182 for 23S rRNA, PX922274–PX922279 for *gltA* and PX922280–PX922289 for *groEL* genes.

Maximum likelihood (ML) analyses based on the 16S rRNA gene showed that the sequences detected in the present study clustered within the genus *Anaplasma* but did not form an isolated monophyletic clade (Figure 1a). ML analyses based on the 23S rRNA gene (Figure 1b) placed the sequences detected in this study within a broader clade closely related to *An. platys*, although with moderate bootstrap support. In contrast, ML analyses based on the ***gltA*** gene (Figure 1c) grouped the sequences obtained in this study into a distinct and well‐supported clade. Similarly, ML analyses based on the ***groEL*** gene (Figure 1d) also clustered the sequences into a distinct clade, phylogenetically related to *An. platys*.

**FIGURE 1:**
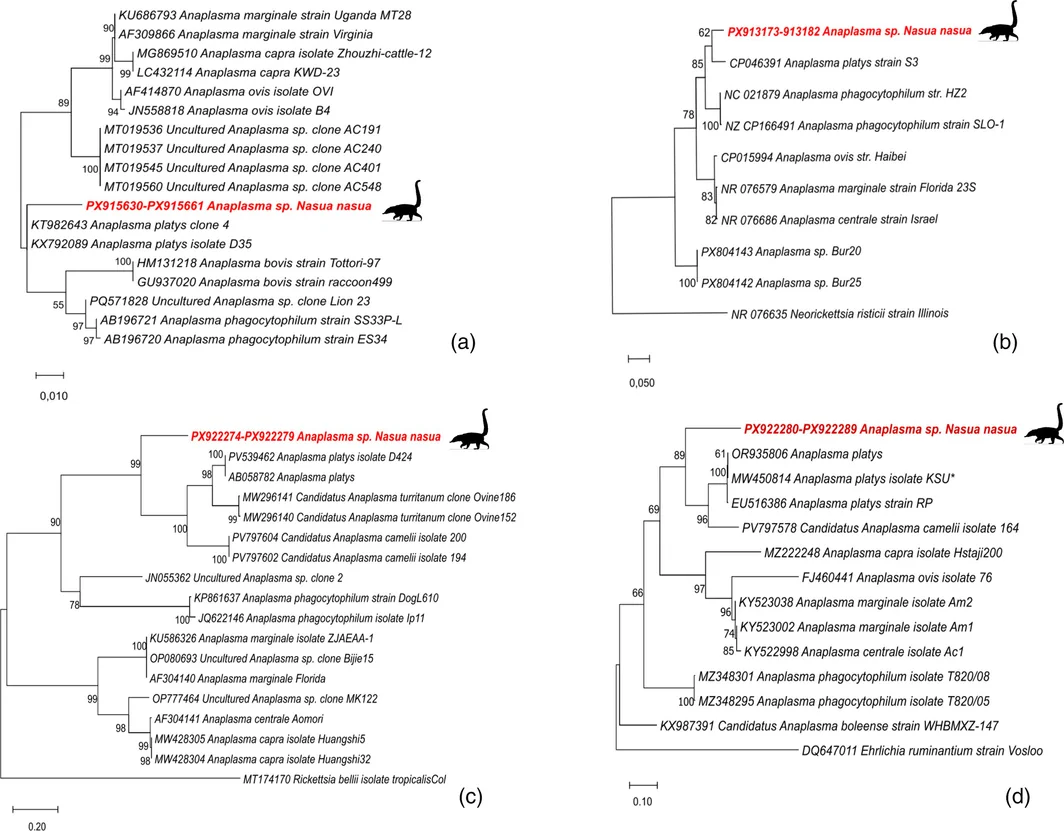
Maximum likelihood phylogenetic trees inferred from partial sequences of different genes. (a) 16S rRNA gene fragment, reconstructed under the Kimura 2‐parameter model with a discrete Gamma distribution to account for rate heterogeneity among sites (5 categories; +G, parameter = 0.1384) and allowing for a proportion of invariable sites (+I, 46.87%). The analysis included 18 nucleotide sequences and 814 aligned positions. (b) 23S rRNA gene fragment, reconstructed using the Kimura 2‐parameter model with rate variation among sites modelled by a discrete Gamma distribution (+G, parameter = 0.2094), including 10 nucleotide sequences and 681 aligned positions. (c) *gltA* gene fragment, reconstructed under the Kimura 2‐parameter model with a discrete Gamma distribution (+G, parameter = 0.7368), based on 18 nucleotide sequences and 415 aligned positions. (d) *groEL* gene fragment, reconstructed using the Tamura 3‐parameter model with a discrete Gamma distribution (+G, parameter = 0.3230), including 14 nucleotide sequences and 574 aligned positions. The scale bar represents the number of substitutions per site. All evolutionary analyses were performed using MEGA v10.2.

Although not the first study to detect arthropod‐borne agents in coatis in the PNI, our study is the first to investigate *Borrelia* spp. infection in coatis in South America and provides novel insights regarding the number of animals analysed (*n* = 73), the frequency of *Anaplasma* spp. positive individuals (47.9%), as well as the fragment length and gene diversity assessed for this pathogen (16S rRNA, 23S rRNA, *gro*EL and *glt*A). Based on our data, we inferred the relationship between positive coatis and the presence of ticks testing either negative or positive for *Anaplasma* spp.

Perles et al. (2023) analysed 49 coatis and reported an *Anaplasma* sp. positivity rate of 14.49%, which is lower than that observed in the present study (47.9%). The sensitivity of the primers used plays a crucial role in pathogen detection. Although nested PCR, as applied by Perles et al. (2023), generally provides greater sensitivity, the oligonucleotides employed may differ in their detection efficiency, as previously demonstrated by Yang et al. (2016).

The samplings conducted by Perles et al. (2023) took place during the same period as the present study and, in some cases, included the same individuals; however, the laboratory analyses were carried out independently. Despite the differences in the primers employed, the 16S rRNA gene fragment amplified by those authors showed 53.7% overlap (450/838 bp) with the fragment sequenced in this study, exhibiting 100% similarity (450/450 bp) across the overlapping region. Additional studies have also reported the presence of *Anaplasma* spp. in coatis. Perles, Moraes, et al. (2022b) identified a positivity rate of 3.6% in *Nasua nasua* from Mato Grosso do Sul, Brazil, within the Cerrado biome. The sequences obtained in this study showed 100% similarity with those generated in the present work, supporting the evidence that this pathogen represents a common haemoparasite in coatis in South America.

Total DNA extracted from ticks infesting animals previously identified as positive for *Anaplasma* sp. did not yield amplification for this agent. Thus, the ticks were not infected with the same *Anaplasma* sp. detected in coatis, just as the coatis did not harbour the *Anaplasma* sp. identified in *Am. coelebs*. The high prevalence of this *Anaplasma* sp. in coatis, but not in ticks, raises questions regarding the origin and host specificity of this microorganism. Although 72 coatis were infested with *Am. coelebs*, only four out of 413 ticks (~1%) carried a distinct *Anaplasma* sp. (Araújo et al., 2023). One might expect that at least one coati would be infected with this agent; however, the absence of detection suggests that this variant does not establish infection in coatis, indicating that they are not competent hosts. This distinction between *Anaplasma* ecotypes, with varying degrees of host compatibility, has been described in several species, including small mammals, reinforcing the concept of non‐competent hosts (Obiegala et al., 2014).

The absence of *Anaplasma* in both these ticks and the coatis they infested suggests the existence of possible alternative transmission routes. In complex wildlife environments, such as the PNI, it is plausible to consider the involvement of other haematophagous arthropods, such as mosquitoes (Diptera: Culicidae), in the transmission of this microorganism. Although there is no direct evidence of *Anaplasma* spp. transmission by mosquitoes, other haemoparasites have been reported to be transmitted by alternative vectors, especially in areas with high host and vector densities (Guo et al., 2016). A global meta‐analysis estimated an average prevalence of 15% for *An. phagocytophilum* in wild reservoirs, suggesting that unconventional ecological cycles may occur across different biomes (Fröhlich et al., 2023; Karshima et al., 2023).

Additionally, recent studies in the Pantanal have revealed a high occurrence of *Anaplasma* genotypes in wild rodents, highlighting the ecological complexity of these agents. For example, Sousa et al. (2017) reported the presence of members of the family Anaplasmataceae in small mammals and their ectoparasites in the Pantanal, including several wild rodents positive for *Anaplasma* spp., indicating the potential involvement of these hosts in non‐classical wildlife cycles. Therefore, future investigations should consider assessing unconventional vectors, as well as seasonal monitoring of parasitaemia in both hosts and local vectors.

It is worth noting that other tick‐borne agents were detected by Araújo et al. (2023), but they were not detected in the coatis infested by the positive ticks. For instance, the authors reported the presence of *Borrelia* sp. in *Am. coelebs*, *Am. ovale* and *Amblyomma* larvae, as well as *Theileria* sp. in *Am. coelebs*; however, none of these agents were detected in the coatis parasitized by these ticks.

Several factors may explain the non‐detection of these agents in mammalian hosts. First, the tick species found infesting coatis are not specific to *N. nasua* and have been reported parasitizing other wild mammals, birds and reptiles, as shown in ecological surveys in the Cerrado (Perles, Moraes, et al., 2022b) and in Atlantic Forest fragments (Araújo et al., 2023; Magalhães‐Matos et al., 2017). In addition, the infection in coatis may have been recent, with parasitaemia levels still below the detection threshold of conventional PCR, or it may represent a chronic infection with a low circulating DNA load, insufficient for amplification. Both scenarios are common limitations in diagnostic studies involving wildlife haemoparasites.

## AUTHOR CONTRIBUTIONS

**Matheus Dias Cordeiro:** Conceptualization; investigation; funding acquisition; writing – original draft; methodology; validation; visualization; writing – review and editing; project administration; supervision; resources; formal analysis; data curation. **Paulo César Magalhães‐Matos:** Conceptualization; investigation; software; visualization; writing – review and editing; project administration; methodology. **Isadora dos Santos Dias:** Investigation; writing – review and editing; resources; data curation. **Jônathan David Ribas Chagas:** Software; methodology; supervision; writing – review and editing; resources; investigation. **Olívia Zen Gianfrancisco:** Investigation; resources; formal analysis; writing – review and editing; visualization. **Ellen Meireles Brandão:** Investigation; writing – review and editing; validation; supervision; resources. **Claudia Bezerra da Silva:** Conceptualization; writing – review and editing; validation; visualization; methodology; supervision. **Bruna de Azevedo Baêta:** Conceptualization; writing – review and editing; methodology; validation; visualization; data curation; supervision; formal analysis.

## CONFLICT OF INTEREST STATEMENT

The authors declare no competing interests.

## ETHICS STATEMENT

This research was carried out after approval by the Ethics Committee for the Use of Animals of the Veterinary Institute of the Federal Rural University of Rio de Janeiro (No. 058/2014 CEUAIV/UFRRJ). The capture of animals, field collection and transport of biological samples were authorized by the Biodiversity Information and Authorization System (SISBio) of the Ministry of the Environment (No. 43614‐3).

## Supporting information


**Table S1.** Sequences of oligonucleotide primers used for the detection of microorganisms of the genera *Borrelia*, *Ehrlichia* and *Anaplasma* and the Piroplasmida order (genera *Babesia* and *Theileria*) in blood of coatis from Foz do Iguaçu, Paraná, Brazil.

**Table S2.** Individual data of 35 coatis (*Nasua nasua*) infected with *Anaplasma* sp. sampled in Iguaçu National Park, Brazil, showing sex, age and tick infestation status.

## Data Availability

The data that support the findings of this study are openly available in NCBI GenBank (https://www.ncbi.nlm.nih.gov/) PX915630–PX915661 for 16S rRNA, PX913173–PX913182 for 23S rRNA, PX922274–PX922279 for gltA and PX922280–PX922289 for groEL genes.
