# Supplementary material for: High prevalence of a host‐associated A naplasma lineage in wild coatis ( N asua nasua) from the Brazilian Atlantic Forest
Source: Med Vet Entomol. 2026 Apr 18;40(4):1087–92. doi: 10.1111/mve.70074 (PMC13749599; doi:10.1111/mve.70074)
Supplement: Supplementary file 1 [file MVE-40-1087-s001.docx]

**Table S2**. Individual data of 35 coatis (*Nasua nasua*) infected with *Anaplasma* sp. sampled in Iguaçu National Park, Brazil, showing sex, age, and tick infestation status.

| **Animal ID** | **Sex** | **Age** | **Tick infestation** |
| --- | --- | --- | --- |
| N1 | Female | Subadult | *Amblyomma* sp*. + Amblyomma coelebs* |
| N2 | Female | Subadult | *A. coelebs* |
| N3 | Female | Adult | *A. coelebs* |
| N5 | Female | Adult | *Amblyomma* sp*. + A. coelebs + Amblyomma brasiliense* |
| N7 | Male | Subadult | *A. coelebs + A. brasiliense* |
| N8 | Male | Subadult | *A. coelebs + A. brasiliense* |
| N9 | Female | Adult | *Amblyomma* sp*. + A. coelebs* |
| N10 | Female | Subadult | *A. coelebs* |
| N11 | Female | Subadult | *Amblyomma* sp*. + A. coelebs + A. brasiliense + Amblyomma ovale* |
| N15 | Female | Adult | *A. coelebs + A. brasiliense + A. ovale* |
| N17 | Male | Subadult | *A. coelebs + A. brasiliense* |
| N30 | Female | Adult | *A. coelebs* |
| N31 | Male | Subadult | *A. coelebs* |
| N34 | Female | Subadult | *A. coelebs* |
| N35 | Female | Adult | *A. coelebs* |
| N38 | Female | Adult | *A. coelebs + A. brasiliense + A. ovale* |
| N40 | Female | Subadult | *A. coelebs* |
| N44 | Male | Adult | *A. coelebs* |
| N45 | Male | Subadult | *A. coelebs* |
| N46 | Female | Subadult | *A. coelebs + A. brasiliense* |
| N48 | Male | Juvenile | *A. coelebs* |
| N49 | Male | Subadult | *A. coelebs* |
| N50 | Male | Juvenile | *A. coelebs* |
| N52 | Female | Juvenile | *A. coelebs* |
| N54 | Female | Juvenile | *A. coelebs + A. brasiliense* |
| N58 | Male | Juvenile | *A. coelebs* |
| N59 | Male | Adult | *A. coelebs + Amblyomma* sp*. + Haemaphysalis* sp*.* |
| N63 | Female | Subadult | *A. coelebs* |
| N64 | Female | Adult | *A. coelebs* |
| N65 | Female | Subadult | *A. coelebs + A. brasiliense* |
| N66 | Female | Subadult | *A. coelebs* |
| N67 | Female | Subadult | *A. coelebs* |
| N68 | Female | Adult | *A. coelebs* |
| N69 | Female | Adult | *A. coelebs* |
| N71 | Female | Adult | *A. coelebs + Amblyomma* sp*.* |
