# Supplementary material for: High prevalence of a host‐associated A naplasma lineage in wild coatis ( N asua nasua) from the Brazilian Atlantic Forest
Source: Med Vet Entomol. 2026 Apr 18;40(4):1087–92. doi: 10.1111/mve.70074 (PMC13749599; doi:10.1111/mve.70074)
Supplement: Supplementary file 2 [file MVE-40-1087-s002.docx]

**Table S1.** Sequences of oligonucleotide primers used for the detection of microorganisms of the genera *Borrelia*, *Ehrlichia*, and *Anaplasma* and the Piroplasmida order (genera *Babesia* and *Theileria*) in blood of coatis from Foz do Iguaçu, Paraná, Brazil.

| **Specificity/bar**  **Gene/Primer name** | **Aim** | **Sequence (5’ - 3’)** | **Amplicon size(bp)** | **References** |
| --- | --- | --- | --- | --- |
| **Anaplasmataceae family** |  |  |  |  |
| **16S rRNA** |  |  |  |  |
| EHR16SD | Screening | GGTACCYACAGAAGAAGTCC | 349 bp | [1] |
| EHR16SR |  | TGCACTCATCGTTTACAG |  |  |
| ***gro*EL gene** |  |  |  |  |
| Ehr-groEL-F | Description | GTTGAAAARACTGATGGTATGCA | 635 bp | [2] |
| Ehr-groEL-R |  | ACACGRTCTTTACGYTCYTTAAC |  |  |
| ***glt*A gene** |  |  |  |  |
| ANA-CS646F | Description | TGCATGCAGATCATGAAC | 421 bp | [3] |
| ANA-CS1076R |  | GAGTAAAARTCAACATTBGG |  |  |
| ***rpo*B gene** |  |  |  |  |
| Ana-rpoBF | Description | GCTGTTCCTAGGCTYTCTTACGCGA | 577 bp | [2] |
| Ana-rpoBR |  | AATCRAGCCAVGAGCCCCTRTAWGG |  |  |
| ***Anaplasma* spp.** |  |  |  |  |
| ***23S rRNA*** |  |  |  |  |
| Ana23S-212f | Description | ATAAGCTGCGGGGAATTGTC | 515 bp | [4] |
| Ana23S-753r |  | TGCAAAAGGTACGCTGTCAC |  |  |
| ***Anaplasma* spp.** |  |  |  |  |
| ***16S rRNA* gene** |  |  |  |  |
| EE-1 | Description | TCCTGGCTCAGAACGAACGCTGGCGGC | 1433 bp | [5] |
| EE-2 |  | AGTCACTGACCCAACCTTAAATGGCTG |  |  |
| EE-3 |  | GTCGAACGGATTATTCTTTATAGCTTGC | 926 bp |  |
| EE-4 |  | CCCTTCCGTTAAGAAGGATCTAATCTCC |  |  |
| ***Ehrlichia* spp.** |  |  |  |  |
| **Dsb** |  |  |  |  |
| DSB-330 | Screening | GATGATGCTTGAAGATATSAAACAAAT | 349 bp | [6] |
| DSB-380 |  | ATTTTTAGRGATTTTCCAATACTTGG |  |  |
| DSB-720 |  | CTATTTTACTTCTTAAAGTTGATAWATC |  |  |
| ***Borrelia* spp.** |  |  |  |  |
| ***fla*B gene** |  |  |  |  |
| FlaLL | Screening | ACATATTCAGATGCAGACAGAGGT | 665 bp | [7] |
| FlaRL |  | GCAATCATAGCCATTGCAGATTGT |  |  |
| FlaLS |  | AACAGCTGAAGAGCTTGGAAT | 354 bp |  |
| FlaRS |  | CTTTGATCACTTATCATTCTAATAGC |  |  |
| **Piroplasmida order** |  |  |  |  |
| **18S rRNA gene** |  |  |  |  |
| BTF1 | Screening | GGCTCATTACAACAGTTATAG | ~950 bp | [8] |
| BTR1 |  | CCCAAAGACTTTGATTTCTCTC |  |  |
| BTF2 |  | CCGTGCTAATTGTAGGGCTAATAC | ~800 bp |  |
| BTR2 |  | GGACTACGACGGTATCTGATCG |  |  |
| **Mammalia phylum** |  |  |  |  |
| ***gapdh* gene** |  |  |  |  |
| GAPDHF | Quality of the extracted DNA | CCTTCATTGACCTCAACTACAT | ~400 bp | [9] |
| GAPDHR |  | CCAAAGTTGTCATGGATGACC |  |  |
| **Ixodidae Family** |  |  |  |  |
| **16S rRNA** |  |  |  |  |
| 16S+ | Quality of the extracted DNA | CCGGTCTGAACTCAGATCAAGT | 460 bp | [10] |
| 16S- |  | GCTCAATGATTTTTTAAATTGCTGT |  |  |

1. Inokuma H, Raoult D, Brouqui P. Detection of *Ehrlichia* *platys* DNA in brown dog ticks (*Rhipicephalus* *sanguineus*) in Okinawa Island, Japan. J. Clin. Microbiol. 38 (2000), 4219–4221. <https://doi.org/10.1128/JCM.38.11.4219-4221.2000>

2 . Dahmani M, Davoust B, Rousseau F, Raoult D, Fenollar F, Mediannikov O. Natural Anaplasmataceae infection in *Rhipicephalus bursa* ticks collected from sheep in the French Basque Country. Ticks Tick Borne Dis. 8 (2017), 18–24. <https://doi.org/10.1016/j.ttbdis.2016.09.009>

3 Inokuma H, Oyamada M, Kelly PJ, Jacobson LA, Fournier PE, Itamoto K, Okuda M, Brouqui P. Molecular detection of a new Anaplasma species closely related to Anaplasma phagocytophilum in canine blood from South Africa. J Clin Microbiol. 43 (2005), 2934-2937. https://doi.org/10.1128/JCM.43.6.2934-2937.2005.

4 Dahmani M, Davoust B, Benterki MS, Fenollar F, Raoult D, Mediannikov O. Development of a new PCR-based assay to detect Anaplasmataceae and the first report of Anaplasma phagocytophilum and Anaplasma platys in cattle from Algeria. Comp Immunol Microbiol Infect Dis. 39 (2015), 39-45. . <https://doi.org/10.1016/j.cimid.2015.02.002>.

5 Barlough JE, Madigan JE, Derock E, Bigornia L. Nested polymerase chain reaction for detection of *Ehrlichia* *equi* genomic DNA in horses and ticks (*Ixodes* *pacificus*). Vet. Parasitol. 63 (1996), 319–329. https://doi.org/10.1016/0304-4017%2895%2900904-3

6 Almeida AP, Souza TD, Marcili A, Labruna MB. Novel *Ehrlichia* and *Hepatozoon* agents infecting the crab-eating fox (*Cerdocyon* *thous*) in southeastern Brazil. J. Med. Entomol. 50 (2013), 640–646. https://doi.org/10.1603/me12272)

7. Stromdahl E, Williamson PC, Kollars TM, Evans SR, Barry R, Vince MA, Dobbs N. Evidence of *Borrelia* *lonestari* DNA in *Amblyomma* *americanum* (Acari: Ixodidae) removed from humans. J. Clin. Microbiol. 41 (2003), 5557–5562. https://doi.org/10.1128/JCM.41.12.5557-5562.2003

8 Jefferies R, Ryan UM, Irwin PJ. PCR-RFLP for the detection and differentiation of the canine piroplasm species and its use with filter paper-based technologies. Vet. Parasitol. 144 (2007), 20–27. https://doi.org/10.1016/j.vetpar.2006.09.022

9 Birkenheuer AJ, Levy MG, Breitschwerdt EB. Development and evaluation of a seminested PCR for detection and differentiation of *Babesia* *gibsoni* (Asian genotype) and *B. canis* DNA in canine blood samples. J. Clin. Microbiol. 41 (2003), 4172–4177. <https://doi.org/10.1128/JCM.41.9.4172-4177.2003>

10. Mangold AJ, Bargues MD, Mas-Coma S. Mitochondrial 16S rDNA sequences and phylogenetic relationships of species of *Rhipicephalus* and other tick genera among Metastriata (Acari: ixodidae). Parasitol Res 1998; 84(6): 478-484. <http://doi.org/10.1007/s004360050433>
